# ONE Health Approach to Address Zoonotic Brucellosis: A Spatiotemporal Associations Study Between Animals and Humans

**DOI:** 10.3389/fvets.2020.00521

**Published:** 2020-09-02

**Authors:** Kun Zhou, Beibei Wu, Hang Pan, Narayan Paudyal, Jize Jiang, Le Zhang, Yan Li, Min Yue

**Affiliations:** ^1^Department of Veterinary Medicine & Institute of Veterinary Sciences, Zhejiang University College of Animal Sciences, Hangzhou, China; ^2^Zhejiang Province Center for Disease Control and Prevention, Hangzhou, China; ^3^Animal Health Research Division, Nepal Agricultural Research Council, Kathmandu, Nepal; ^4^College of Computer Science, Sichuan University, Chengdu, China; ^5^Zhejiang Provincial Laboratory of Preventive Veterinary Medicine, Hangzhou, China

**Keywords:** *Brucella*, brucellosis, China, spatial, temporal

## Abstract

**Background:** Brucellosis is one of the most significant zoonosis over the world, threatening both veterinary and human public health. However, few studies were focused on nationwide animal brucellosis and made association with human brucellosis.

**Methodology and Principal Findings:** We conducted a bilingual literature search on *Brucella* or brucellosis in China on the two largest databases (China National Knowledge Infrastructure and PubMed) and conducted a systematic review. A total of 1,383 Chinese and 81 English publications, published between 1958 and 2018 were identified. From them, 357 publications presenting 692 datasets were subjected to the meta-analysis. The overall prevalence rate is 1.70% (95% CI: 1.66–1.74), with a declining (until the late 1990s) and rising trend (starting the early 2000s). Interestingly, the animal with highest prevalence rate is canine (8.35%, 95% CI: 7.21–9.50), and lowest in cattle (1.22%, 95% CI: 1.17–1.28). The prevalence of *Brucella* in animals was unequally distributed among the 24 examined regions in China.

**Conclusions:** Brucellosis is a reemerging disease for both humans and animals in China. The observed data suggests that dogs and yaks are the leading reservoirs for *Brucella*, and the provinces with highest prevalence rates in animals are Hubei, Sichuan, Inner Mongolia, Fujian, and Guizhou. Accordingly targeted intervention policy should be implemented to break the *Brucella* transmission chain between animals and humans in China.

## Introduction

Zoonotic diseases are capable of infecting humans from animals by direct contact, or via food, water and environment, representing a significant public health problem. Brucellosis, caused by multiple species of the genera *Brucella*, is a textbook example of zoonotic disease prevalent all over the world. Brucellosis, recognized as the leading neglected zoonotic diseases by World Health Organization (WHO), remains a major epidemic, particularly in low- and middle-income countries, including China (1–4). During the past decades, it was observed a significant increase in human brucellosis in China (5, 6). Given a broad range of animal reservoirs, and humans serving as an accidental host, the exact relationship between human brucellosis and its animal reservoirs remains elusive. This is one of the most pressing knowledge gaps to understand the dynamics of zoonotic *Brucella* in China.

The *Brucella* pathogens infect and invade the host via the mucous membranes, gastrointestinal tract, respiratory tract or abraded skin. In China, human infections are more often related to the occupation and consumption of foods of animal origin (7). Veterinary personnel, animal-farmers, and slaughterhouse workers are at the highest risk, usually acquiring infections through the mucous or abraded skin (8, 9). Respiratory tract infections are common in people inhaling contaminated air-borne droplets or dust, for example, those working in the fur processing environment (10). People with no exposure to farm animals or animal-products are usually infected by contaminated food or water via gastrointestinal tract infections (11). The disease burden of human brucellosis infections is dynamic with respect to the endemic areas and time. Despite this dynamism, the only static component in the transmission chain is that the human incidences of brucellosis are always linked to certain animal reservoirs. Here, for the first time, we can address these key questions by using a “One Health” approach in an interconnected manner (6).

Farm animals, companion animals, and wild animals are well-known reservoirs for human *Brucella* infections (10). Farm animal brucellosis, with a wide range of clinical signs, is dependent on the infected animal hosts, age, sex, routes of exposure and organs involved (12). The hallmarks of infection include spontaneous abortion, undulant fever, and sometimes mastitis. Generally, animal brucellosis is sub-clinical but causes significant economic losses in the livestock industry. In companion animals, most of the infection cases are usually asymptomatic or mild (13). Therefore, farm and companion animal brucellosis are usually underappreciated due to their obscure syndrome and clinical presentations. Nevertheless, a systematic understanding the burden of animal brucellosis is largely lacking in China.

In this study, we conducted a systematic review and meta-analysis of the spatiotemporal epidemiological feature of brucellosis in animals and humans from a nationwide One Health approach. A comprehensive review of animal brucellosis in China, with an integrated evidence-based result analysis of human brucellosis to address the parameters for potential transboundary infections, was also undertaken.

## Materials and Methods

### Search Strategy

This review and systematic analysis included studies published in Chinese or English languages, between 1958 and 2018. The Chinese Publications were retrieved from China National Knowledge Infrastructure, CNKI (https://www.cnki.net/) and English papers were from PubMed with the same search string composed using keywords “*Brucella* or Brucellosis” and “China.”

### Selection Criteria

Publications were firstly screened based on their title and abstract followed by inclusion or exclusion criteria formulated by the authors (Table 1).

**Table 1:** Inclusion/exclusion criteria for publication screening.

**Inclusion criteria**	**Exclusion criteria**
• Case report on brucellosis	• Literature review
• Epidemiological investigation of brucellosis	• Publication with no positive sample
• Study on biotype of *Brucella* strains	• Number of samples is uncertain
	• No Chinese strains or cases
	• Article with repeated data
	• Study not related to *Brucella* or brucellosis
	• Full text not available
	• Sample type and province are not available

### Data Extraction

Selected publications were perused for data extraction. Information on the title, author, journal, publication year, province, host (sheep and goat were grouped into one category because they were referred to as one single entity in Chinese literature), sampling year, type of samples, number of total samples, number of positive samples and number of *Brucella* strains and species wherever available, were determined. That information was extracted and arranged in an MS Excel spreadsheet formatted especially for this purpose.

### Descriptive Analysis

Descriptive analysis was undertaken to calculate an approximately accurate epidemiologic picture of the disease and its causative agent. We analyzed the spatiotemporal relationship between human and animal brucellosis, where possible. We identified dominant species of *Brucella* in humans and some common animals including cattle, dog, pig, sheep & goat, and pointed out potentially high-risk infectious sources toward the human.

### Meta-Analysis

A second step quality control screening of each record used for the descriptive analysis was applied using the exclusion criteria (Table 2). This was used to revise the data necessary for a meta-analysis of the prevalence of animal brucellosis.

**Table 2:** Exclusion criteria for the second round of record screening.

1. No specific host or host mentioned is human
2. No specific sample types mentioned
3. Total number of samples is less than 100
4. No specific province reported in the study
5. No reporting of any specific sampling year

The data extracted from hence selected publications were meta-analyzed in a binary random-effects model by DerSimonian-Laird method at 95% confidence level using OpenMeta-analysis, as described previously (14). The meta-analysis was performed according to the Preferred Reporting Items for Systematic Reviews and Meta-Analyses (PRISMA) guidelines to ensure the quality of the writing and presentation of this review (15). Three subgroup meta-analysis on the host, sampling year, and province were conducted simultaneously. The results obtained as the forest plots were projected with GraphPad Prism 7.

## Results

### Literature Search and Data Extraction

**Figure 1:**
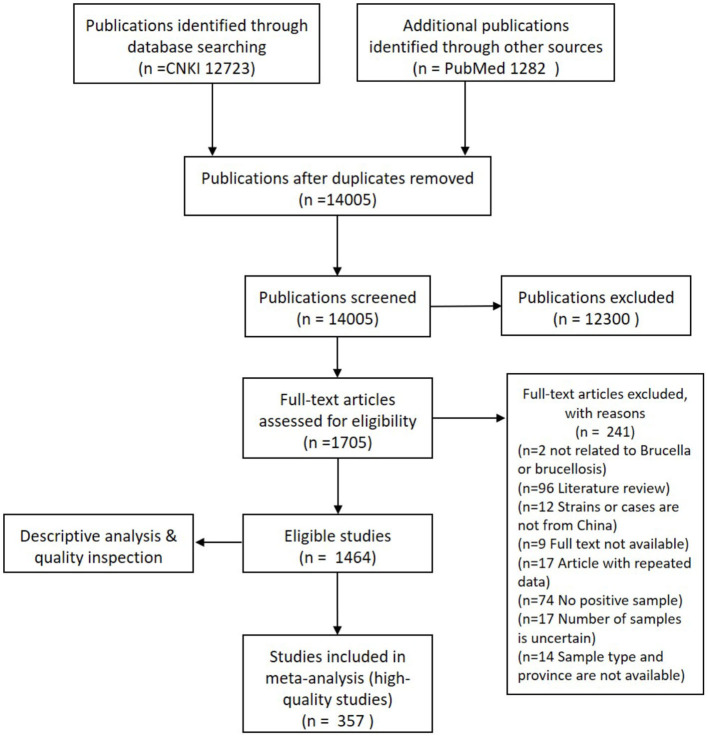
A flow chart of literature research and data extraction.

A total of 1,464 bilingual publications, which included 1,383 in Chinese and 81 in English, were used for the descriptive analysis after a full-text screening of primary eligible studies. Only 357 publications, 350 in Chinese, and 7 in English, among the total of 692 eligible records, were deemed suitable for meta-analysis. A flow chart of literature research and data extraction is shown (Figure 1).

### Descriptive Analysis

All provinces or municipality cities in China except Macao had reported positively for the prevalence of brucellosis. In mainland China, the largest number of records were from Inner Mongolia (*n* = 431), while there were only a few records from Tianjin (*n* = 11). Rose-Bengal Plate Agglutination Test (RBPT), Standard Tube Agglutination Test (SAT), Polymerase Chain Reaction (PCR), and Enzyme-Linked Immunosorbent Assay (ELISA) were the most common assays used for the detection of positive samples which were mostly blood. A total of 70,035,273 samples were tested in all these eligible studies, while 2,339,773 samples turned out to be positive, considered as the confirmed brucellosis cases or separated strains in these studies. There were more records on *Brucella* and brucellosis in northern China than in southern China. The number of samples tested in northern China was also much higher. The details are shown in Table 3 below (Some data are not counted into the table because they are lacking information either on host or on sampling places).

**Table 3:** Characteristics of records from eligible studies.

	**Total**		Northern China^*^		**Southern China^#^**	
	**Human**	**Animal**	**Human**	**Animal**	**Human**	**Animal**
Number of records (%)	1,904	1,382	1,386 (73%)	939 (68%)	480 (25%)	409 (30%)
Number of total samples (%)	10,283,133	59,741,718	8,522,768 (83%)	55,531,284 (93%)	1,360,477 (13%)	2,668,094 (4%)
Number of positive samples (%)	1,037,606	1,293,024	919,372 (89%)	1,202,177 (93%)	24,156 (2%)	78,523 (6%)

* *Northern China: Includes provinces such as Heilongjiang, Jilin, Liaoning, Inner Mongolia, Gansu, Xinjiang, Qinghai, Ningxia, Shanxi, Shaanxi, Hebei, Beijing, Tianjin, Shandong, Henan*.

# *Southern China: Includes provinces such as Shanghai, Jiangsu, Anhui, Hubei, Chongqing, Sichuan, Yunnan, Guizhou, Hunan, Jiangxi, Zhejiang, Fujiang, Taiwan, Guangzhou, Hong Kong, Guangxi, Hainan, Tibet*.

**Figure 2:**
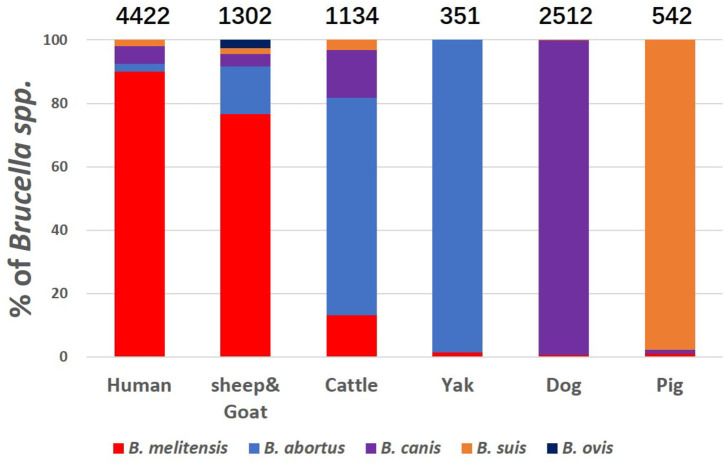
Prevalence of various species of *Brucella* on different hosts. Height of the column in different color shows the proportion of *Brucella* species. Hosts are specified as human, sheep & goat, cattle, yak, dog, and pig at the base of each bar. Number above the bars indicates the number of positive samples.

We also calculated the number of specific *Brucella* species mentioned in all these studies in human, sheep & goat, cattle, yak, dog and pig. Only five species, *B. melitensis, B. canis, B. abortus, B. suis*, and *B. ovis* were detected (Figure 2). Humans are most likely to be infected by all the four *Brucella* species from all kinds of animal host albeit in varying proportions. The proportion of presence of these pathogens in humans and sheep & goat are highly similar, illustrating that sheep & goat may be the likely source of infection for human brucellosis. *Brucella* serovars infecting yak, dog and pig are apparently highly species-specific, for example, *B. abortus* for yak*, B. canis* for dog, and *B. suis* for pig.

**Figure 3:**
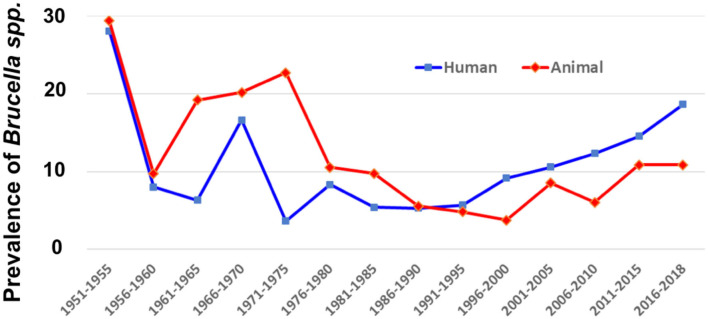
Temporal distribution of human and animal brucellosis in China between 1951 and 2018.

**Figure 4:**
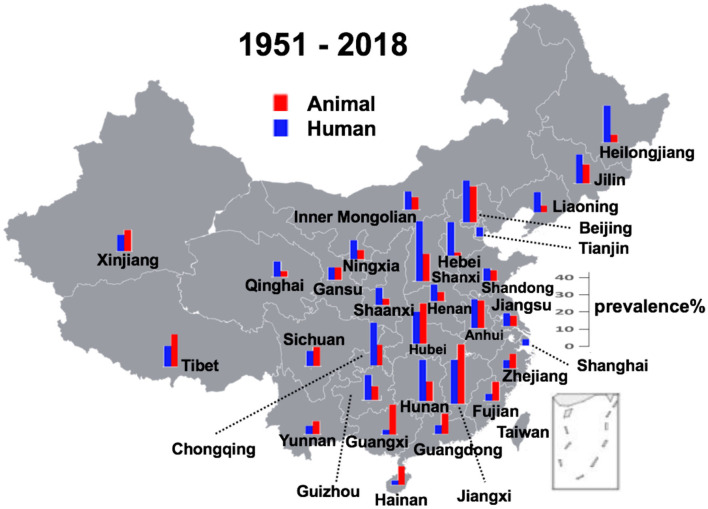
Geographical distribution of brucellosis in animal reservoirs and humans. In two municipal cities Shanghai and Tianjin, only human cases were observed.

The average prevalence rates of all the records were calculated and the results are illustrated as the temporal (Figure 3) and spatial (Figure 4) prevalence rates in humans and animals in China. Prevalence rates of human and animal brucellosis both have a decreasing trend, until the late 1990s, followed by an increasing trend, starting the early 2000s. Interestingly, prevalence of brucellosis in animals was higher than that of humans before the year 1995, while after 1995 the prevalence of brucellosis in humans becomes higher.

Provinces in southern China had a higher prevalence rate before 1990, and currently, central Chinese provinces make the lead. There is always a higher rate of prevalence in the animals than in humans in most littoral areas, while things are on the contrary in the inwards areas. Regarding the geographic distribution, except in two municipal cities of Shanghai and Tianjin where only human cases were recorded, all other provinces had recorded an almost simultaneous increase or decrease in the prevalence in human and animal subjects. The analysis also reveals that the prevalence in hosts was high before 1990, then it started a downhill trend from 1991 to 2000, but again climbing up with a gradual increase after the 2000s at multiple geographical regions in mainland China. This spatiotemporal dynamism for human and animal brucellosis in China from 1951 to 2018 has been clearly shown (Supplemental Figure 1).

### Prevalence of Brucellosis in Animals and Humans

A total of 357 publications screened twice (using the criteria as given in Table 2) were put into a meta-analysis, followed by a subgroup analysis on three variables including sampling year, geographical regions and host was also undertaken. Subgroups with less than five records were also put into meta-analysis but were not showed in the results below.

**Figure 5:**
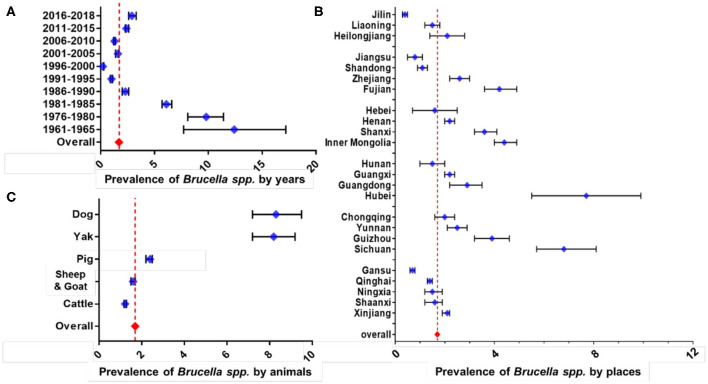
Results of subgroup meta-analysis for different animal-related factors. Subgroup analysis on sampling year **(A)**, subgroup analysis on the province **(B)**, and subgroup analysis on the host **(C)**. The broken vertical line indicates the pooled summary estimate of prevalence for the selected variable.

The overall prevalence rate among all animals in China from 1951 to 2018 was estimated to be 1.70% (95% CI: 1.66–1.74) (Figure 5A). As a factor of time, the largest number of records were for years between 2011 and 2015 (*n* = 170), whereas the smallest was for 1961–1965 (*n* = 6). There was an insufficient number of records (less than five) for the years 1951–1955, 1956–1960, 1966–1970, and 1971–1975 so these time frames were not shown. The result of subgroup analysis on the sampling year showed declining followed by a rising trend. Period with the lowest prevalence rate, irrespective of the host was 0.24% during 1996–2000 (63 records, 95% CI: 0.20–0.27), while the one with the highest prevalence was 12.44% during 1961–1965 (6 records, 95% CI: 7.70–17.19). In recent years, 2016–2018 the prevalence rate was 2.91% (29 records, 95% CI: 2.57–3.26), ~10 times higher than the lowest one.

There were only 24 provinces/autonomous regions which had no less than five qualified datasets for meta-analysis (Figure 5B). Five provinces/autonomous with the highest rate were Hubei (5 records, 7.69%, 95% CI: 5.51–9.87), Sichuan (20 records, 6.86%, 95% CI: 5.68–8.04), Inner Mongolia (50 records, 4.44%, 95% CI: 4.04–4.85), Fujian (20 records, 4.25%, 95% CI: 3.58–4.92), and Guizhou (18 records, 3.89%, 95% CI: 3.18–4.59). In contrast, five lowest rates were for Jilin (16 records, 0.39%, 95% CI: 0.29–0.48), Gansu (55 records, 0.70%, 95% CI: 0.60–0.79), Jiangsu (15 records, 0.77%, 95% CI: 0.49–1.05), Shandong (37 records, 1.13%, 95% CI: 0.95–1.31), and Qinghai (112 records, 1.40%, 95% CI: 1.29–1.52).

Among the five different types of host groups analyzed (Figure 5C), dog possessed the highest prevalence rate (52 records, 8.35%, 95% CI: 7.21–9.50), followed by yak with 43 records and 8.22% prevalence rate (95% CI: 7.23–9.20), while cattle (228 records, 1.22%, 95% CI: 1.17–1.28), sheep & goat (277 records, 1.61%, 95% CI: 1.54–1.67), and lastly the pigs (63 records, 2.35%, 95% CI: 2.16–2.54). This preferably indicates that dogs and yaks could be the emerging host that possess some significant threat to *Brucella* transmission in China. The detailed results of subgroup analysis are shown as forest plots in Figures 5A–C.

## Discussion

Brucellosis is one of the most common zoonotic diseases worldwide, with a bioweapon potential, posing a significant public health concern (16). It not only causes considerable damages to public health but also significantly impairs veterinary public health and animal welfare. For example, reduced milk yield and contaminated dairy products due to animal brucellosis in America alone worth ~400 million US dollars (17), and the annual economic losses of brucellosis in two counties in Jilin province, China was more than 2.3 million US dollars (18). Brucellosis had been well-controlled last century in China by a nationwide program for brucellosis control, as well as by a national campaign for preventing and controlling brucellosis (19). In China, it had been well-controlled by a national campaign for preventing and controlling brucellosis in the last century. However, the disease has reemerged during the last two decades (20). In certain regions of China, the number of cases of brucellosis reported is higher than that in the 1990s (21). Recently, a study on the epidemiology of human brucellosis was carried out with official data (6). Moreover, Ran and his group applied a meta-analysis on seroprevalence in dairy cows in China (22). Despite these interesting and novel studies, information on the dominant biotype of *Brucella* in multiple kinds of hosts and prevalence within other animals is ambiguous. To a general extension, our unique One Health approach, combined with evidence-based meta-analysis, could be readily scaled to other zoonotic agents with significant public health importance.

In China, brucellosis was first recorded as Malta fever in two foreigners in Shanghai in 1905 (6). Until the 1990s, there was a high incidence of animal brucellosis, with relatively few human incidence (Supplemental Figure 1). Then after the 1990s, it started an uphill climb in most of the regions of mainland China. Brucellosis, previously thought to be frequently detected in northern China, is now increasingly seen in highly cosmopolitan parts of southern China (23). Our findings are in line with an earlier study which, using the notifiable reporting data for 1955–2014 based on the magnitude and distribution of human brucellosis in mainland China, had emphasized its recent re-emergence (6). Similar to the findings of our analysis, a previous study focusing only on the human incidence of brucellosis, showed some distinct temporal patterns, such as high incidence during 1955–1978, low incidence during 1979–1994, and dramatic accumulation incidences from 1995 onwards is consistent with the trend in *Brucella* seroprevalence from animal and human sero-surveys conducted in China during 1950–2014 (6). In general, where human prevalence of brucellosis is higher than prevalence of brucellosis in animals are mainly in southern China, including Hainan, Guangxi, Guangdong, Fujian, Jiangxi, Zhejiang, and Hubei (Figure 4). This might be because (1) a lack of animal surveillance capabilities in southern regions as a result of small population of large livestock; (2) the imported animal food contaminated with *Brucella* are responsible for the infections, which further support the multiple routes for disease transmission (24–28). These reasons might be lead to extreme cases in particular years and places where there is only human brucellosis without an indication of animal brucellosis (Supplemental Figure 1).

In China, 90% of brucellosis occurs in six northern agricultural provinces including Inner Mongolia, Shanxi, Heilongjiang, Hebei, Jilin, and Shaanxi. The Inner Mongolia Autonomous Region has the highest incidence rate of human and animal brucellosis throughout China (29). Human cases have been observed in those regions where animal farming is prevalent, particularly in areas with many poor rural farms and pastures in northwest China (30). However, it is suggested that there is a change in the epidemiology of brucellosis in China (23). Recent publications show that the areas of brucellosis endemicity gradually shifted from pasturing areas, i.e., Inner Mongolia, Xinjiang, Tibet, Qinghai, and Ningxia, to grassland and agricultural areas, i.e., Shanxi, Liaoning, Hebei, Shandong, and Jilin provinces, and the southern provinces became increasingly affected (6). Some analyses further revealed that patients from Guangdong province were more likely to have consumption of apparently exotic foods such as goat placenta (23), thereby manifesting as increased incidence of brucellosis. Although incidence appeared to increase in every province, Tibet seems to follow an inverse pattern and reported a few cases during the past 10 years (6). These hint on the probable movement of infected animals or contaminated food from northern to southern China and thus may explain the current change in the epidemiology of brucellosis in China (23).

Sheep and goats are the major herbivores in northwest China, and they are primarily kept by poor rural farmers in pastoral areas. In general, a considerable number of sheep and yaks are raised on the same grazing land on the Tibetan plateau, and these animals are considered to be the main hosts of *Brucella* (30). Brucellosis was recognized in Tibet in the 1980s as a disease of both livestock and humans. Some study indicates that bovine brucellosis is endemic among the yak population in the plateau region of China (31).

In the last 12 years, brucellosis has re-emerged in most regions of China, showing an annual accumulation in animal and human infections during this period (29). This is also evident in our meta-analysis which shows a general increasing trend in the prevalence, particularly for the recent past years. With regard to the geographic distribution, our investigation confirmed that sero-prevalence in eastern regions was higher than the central and western regions. This conclusion is also supported by the localized and dense clusters of small ruminants in those regions.

Our analysis has shown that most of the human cases of brucellosis were caused by *B. melitensis*. It is well-known that *B. melitensis* is a dominant epidemic strain in animal and *B. abortus* and *B. suis* can also infect sheep; *B. suis* biovar 3, especially emerged in Inner Mongolia (32). *B. melitensis* genotype ST8 was not only the predominant genotype in sheep but also responsible for human brucellosis (33). The results of seroprevalence assays have confirmed that *Brucella* spp. was mainly epidemic in sheep and dairy cows, whereas other animals had a very low incidence. These results also showed a strong host preference for certain *Brucella* species. Furthermore, this also indicates that small ruminants or cattle are the primary risk factors for human contagion. Human brucellosis in northwest China is closely related to infectious sheep. From an epidemiological perspective, the major causes of brucellosis in animals in China have been sheep infected by *B. melitensis* (34). Sheep and dairy cows with *Brucella* had higher infection trends, although the positive rates in dairy cows dipped to a downward trend between 2013 and 2014. Compared with this, in sheep and dairy cows, brucellosis in yellow cattle and dogs maintained sporadic onset, and no cases were found in swine (34).

A notable feature in our analysis is the presence of *B. canis* in all other hosts except for pig and yak. Human brucellosis caused by *B. suis* and *B. canis* in Guangxi, China (35) have also been reported earlier. An earlier study reports that *B. canis* is a rare source of human brucellosis in China (36), where *B. melitensis* has been the major pathogen associated with human brucellosis outbreaks. *B. canis* infection was first reported in China in 1983 (13), but now our data indicates that this has become the leading reservoir for emerging brucellosis. This scenario will be even more challenging, considering a significantly growing population in canine market. In a less frequent situation, *B. suis* can also be directly or indirectly transferred from swine to sheep, which could act as reservoirs for *B. suis* infection and later transmitted to humans (37). Therefore, an enhanced surveillance system for companion animals in the densely populated cities is urgently needed.

Our data and subsequent analysis have thus revealed that brucellosis was a major concern for study and research in Northern China as compared to Southern China over time, for around three-quarters publications on brucellosis were carried out in North China. However, the meta-analysis showed that some areas in Southern China had even higher prevalence rates than northern ones, suggesting that brucellosis in the south is as severe as the north. It can be inferred from these analyses that human is likely to get infected with *Brucella* from all available transmission pathways, and various kinds of animals, among which the sheep and goat are possibly the highest risks because the formation of *Brucella* species in sheep and goat is similar with that of human. More alarmingly, canine brucellosis could be a top public health concern imposing a greater threat to human because pet dogs are playing a more and more important role in the current days of Chinese life. According to statistics, there have been over 740 million domestic dogs in China in 2019 (38), relationships between pet dogs and human are getting more and more close, which emphasizes that canine brucellosis is a pressing concern. An earlier study had shown that Chinese isolates of *B. suis* strains had unique genetic lineages at the global level whereas the isolates of *B. canis* were closely homologous to strains from Korea (35). Despite this disease being the most widespread zoonosis globally, it is also true that it remains severely neglected as a potential cause for chronic, debilitating maladies for multiple reasons such as its non-descript clinical presentation in human populations and its varying degree of clinical presentations (11).

Despite our updated largest analysis, there are some limitations to our analysis. Firstly, we only used two databases for bilingual publication research, so we might have missed some literatures. Second, most publications chose RBPT and SAT to detect positive blood samples, which have lower specificity and sensitivity than other recommended methods such as ELISA. Only 24 provinces in China had sufficient qualified data and thus were eligible for meta-analysis, leaving a quite large leak of animal brucellosis in remaining regions in China. Despite these, owing to a large number in the examined dataset, we believe that our meta-analysis is able to unravel a general situation and trend of animal brucellosis in China over time, thus attracting more attention to prevention and control of brucellosis in China.

## Conclusion

Brucellosis is an important zoonosis in China, which was controlled well in the late twentieth century but re-emerged in the past two decades. Human may get infected from various domesticated animals, and most possibly sheep and goat. Canine brucellosis deserves more attention for the prevalence rate in dogs are quite high and the relationship between human and dogs is more and more close. Studies on brucellosis and *Brucella* were gaining more attention at northern provinces than those of southern region. However, our findings suggests that brucellosis among animals in the south is as equally serious as that in the north. Therefore, a nationwide comprehensive monitoring program must be reinitiated for both human and animals. In this perspective, brucellosis being a zoonotic disease, the One Health approach would be an ideal tool for alleviating the other diseases impacts on both humans and animals with the public health concerns.

## Author Summary

Brucellosis is a worldwide zoonosis caused by *Brucella* posing significant damage to both public health and agriculture. However, the report about animal brucellosis in China is scattered, unintegrated, and dominant species among each kind of animals remains unknown. Here, we aimed to present the nationwide evidence-based results by using the systematic review and meta-analytic approach.

## Evidence Before This Study

After over 100 years in discovery of the agent of Malta fever by David Bruce, brucellosis remains one of the important zoonotic infections causing huge reproductive failure in livestock, and economic losses of the dairy industry in low and middle-income countries. Brucellosis is also a serious public health problem for humans, due to food-chain, and/or occupational exposure. It is estimated that over one-fifth of the 1.4 billion worldwide cattle population is currently infected by *Brucella*, and results in over half-million new human cases annually, representing the most common systemic bacterial zoonosis. As the world largest population (1.4 billion people and 500 million livestock units), even though numerous studies reported significant accumulation of human brucellosis in China, only scattered and unintegrated animal brucellosis datasets are available, leaving huge knowledge gap for the zoonotic control and measurement.

## Added-Value of This Study

We used the evidence-based studies, a meta-analysis, to build the most updated and comprehensive picture of brucellosis in China, including humans, livestock, and companion animals. The One Health approach indicates that the trend of human and animal brucellosis are closely associated with each other in China, with significant expansion from traditional northern farming areas to southern China. Importantly, we found that canine and yak could serve as the emerging reservoirs for human infections in China.

## Implications of All The Available Evidence

The surveillance measurement for human brucellosis should be shifted from traditional northern China to southern regions. Policymakers should consider regional epidemiological data and risk factors to mitigate the zoonotic transmission from animals to humans. Our unique One Health approach, combined with evidence-based meta-analysis, could be readily scaled to other zoonotic agents with significant public health importance.

## Data Availability Statement

All datasets generated for this study are included in the article/Supplementary Material.

## Author Contributions

MY: conceptualization. KZ and BW: data curation. KZ and NP: formal analysis and visualization. MY, NP, and HP: methodology. MY and YL: project administration, and writing—review and editing. NP, HP, and JJ: software. MY, YL, and LZ: supervision. KZ: writing—original draft. All authors contributed to the article and approved the submitted version.

## Conflict of Interest

The authors declare that the research was conducted in the absence of any commercial or financial relationships that could be construed as a potential conflict of interest.

## Supplementary Material

Supplemental Figure 1 Spatiotemporal prevalence of human and animal brucellosis in China from 1951 to 2018.

## References

[1] Rubach MP Halliday JE Cleaveland S Crump JA. Brucellosis in low-income and middle-income countries. Curr Opin Infect Dis. (2013) 26:404–12. 10.1097/QCO.0b013e3283638104 23963260 PMC3888775

[2] Pappas G Papadimitriou P Akritidis N Christou L Tsianos EV. The new global map of human brucellosis. Lancet Infect Dis. (2006) 6:91–9. 10.1016/S1473-3099(06)70382-6 16439329

[3] Musallam II Abo-Shehada MN Hegazy YM Holt HR Guitian FJ. Systematic review of brucellosis in the Middle East: disease frequency in ruminants and humans and risk factors for human infection. Epidemiol Infect. (2016) 144:671–85. 10.1017/S0950268815002575 26508323

[4] Cárdenas L Awada L Tizzani P Cáceres P Casal J. Characterization and evolution of countries affected by bovine brucellosis (1996-2014). Transbound Emerg Dis. (2019) 66:1280–90. 10.1111/tbed.13144 30739391

[5] Kong W. Brucellosis infection increasing in Southern China. Eur J Intern Med. (2018) 51:e16–8. 10.1016/j.ejim.2018.03.004 29549979

[6] Lai S Zhou H Xiong W Gilbert M Huang Z Yu J. Changing epidemiology of human brucellosis, China, 1955-2014. Emerg Infect Dis. (2017) 23:184–94. 10.3201/eid2302.151710 28098531 PMC5324817

[7] Xiao Y Zou G Yin J Tan W Zhou J Zhang H. Seroepidemiology of human *Brucella* infection in Yixing, China. Trop Doc. (2017) 47:165–7. 10.1177/0049475516640191 27079490

[8] Krueger WS Lucero NE Brower A Heil GL Gray GC. Evidence for unapparent *Brucella canis* infections among adults with occupational exposure to dogs. Zoonoses Public Health. (2014) 61:509–18. 10.1111/zph.12102 24751191

[9] Lytras T Danis K Dounias G. Incidence patterns and occupational risk factors of human brucellosis in Greece, 2004-2015. Int J Occup Environ Med. (2016) 7:221–6. 10.15171/ijoem.2016.806 27651083 PMC6817955

[10] Mangalgi SS Sajjan AG Mohite ST Gajul S. Brucellosis in occupationally exposed groups. J Clin Diagn Res. (2016) 10: Dc24–7. 10.7860/JCDR/2016/15276.7673 27190804 PMC4866102

[11] Hull NC Schumaker BA. Comparisons of brucellosis between human and veterinary medicine. Infect Ecol Epidemiol. (2018) 8:1500846. 10.1080/20008686.2018.1500846 30083304 PMC6063340

[12] Deng Y Liu X Duan K Peng Q. Research progress on brucellosis. Curr Med Chem. (2019) 26:5598–608. 10.2174/0929867325666180510125009 29745323

[13] Piao D Wang H Di D Tian G Luo J Gao W. MLVA and LPS characteristics of *Brucella canis* isolated from humans and dogs in Zhejiang, China. Front Vet Sci. (2017) 4:223. 10.3389/fvets.2017.00223 29326956 PMC5741857

[14] Paudyal N Pan H Liao X Zhang X Li X Fang W. A meta-analysis of major foodborne pathogens in Chinese food commodities between 2006 and 2016. Foodborne Pathog Dis. (2018) 15:187–97. 10.1089/fpd.2017.2417 29652195

[15] Shamseer L Moher D Clarke M Ghersi D Liberati A Petticrew M. Preferred reporting items for systematic review and meta-analysis protocols (PRISMA-P) 2015: elaboration and explanation. BMJ. (2015) 350:g7647. 10.1136/bmj.g7647 25555855

[16] Ariza J Bosilkovski M Cascio A Colmenero JD Corbel MJ Falagas ME. Perspectives for the treatment of brucellosis in the 21st century: the ioannina recommendations. PLoS Med. (2007) 4:e317. 10.1371/journal.pmed.0040317 18162038 PMC2222927

[17] Seleem MN Boyle SM Sriranganathan N. Brucellosis: a re-emerging zoonosis. Vet Microbiol. (2010) 140:392–8. 10.1016/j.vetmic.2009.06.021 19604656

[18] Liu F Wang D Wang J Li T Zhao Y Jiang S National brucellosis intervention pilot county survey on the economic losses. Chin J Control Endemic Dis. (2008) 23:424–5.

[19] Shang D 50-Year research on prevention and control of brucellosis in China. Chin J Epidemiol. (2000) 21:55–57. 15460005

[20] Jia P Joyner A. Human brucellosis occurrences in Inner Mongolia, China: a spatio-temporal distribution and ecological niche modeling approach. BMC Infect Dis. (2015) 15:36. 10.1186/s12879-015-0763-9 25644986 PMC4319220

[21] Zhang J Yin F Zhang T Yang C Zhang X Feng Z. Spatial analysis on human brucellosis incidence in mainland China: 2004-2010. BMJ Open. (2014) 4:e004470. 10.1136/bmjopen-2013-004470 24713215 PMC3987733

[22] Ran X Cheng J Wang M Chen X Wang H Ge Y. Brucellosis seroprevalence in dairy cattle in China during 2008-2018: a systematic review and meta-analysis. Acta Trop. (2019) 189:117–23. 10.1016/j.actatropica.2018.10.002 30308207

[23] Ye HY Xing FF Yang J Lo SK Lau RW Chen JH. High index of suspicion for brucellosis in a highly cosmopolitan city in southern China. BMC Infect Dis. (2020) 20:22. 10.1186/s12879-019-4748-y 31914937 PMC6950854

[24] Pan H Paudyal N Li X Fang W Yue M. Multiple food-animal-borne route in transmission of antibiotic-resistant salmonella newport to humans. Front Microbiol. (2018) 9:23. 10.3389/fmicb.2018.00023 29410657 PMC5787089

[25] Pan H Zhou X Chai W Paudyal N Li S Zhou X. Diversified sources for human infections by *Salmonella enterica* serovar newport. Transbound Emerg Dis. (2019) 66:1044–8. 10.1111/tbed.13099 30548172 PMC6634944

[26] Wang X Biswas S Paudyal N Pan H Li X Fang W. Antibiotic resistance in salmonella typhimurium isolates recovered from the food chain through national antimicrobial resistance monitoring system between 1996 and 2016. Front Microbiol. (2019) 10:985. 10.3389/fmicb.2019.00985 31134024 PMC6514237

[27] Elbediwi M Pan H Biswas S Li Y Yue M. Emerging colistin resistance in *Salmonella enterica* serovar Newport isolates from human infections. Emerg Microbes Infect. (2020) 9:535–8. 10.1080/22221751.2020.1733439 32122270 PMC7067173

[28] Paudyal N Pan H Wu B Zhou X Zhou X Chai W. Persistent asymptomatic human infections by *Salmonella enterica* serovar newport in China. mSphere. (2020) 5:e00163-20. 10.1128/mSphere.00163-20 32461269 PMC7253594

[29] Liu ZG Wang M Ta N Fang MG Mi JC Yu RP. Seroprevalence of human brucellosis and molecular characteristics of *Brucella* strains in Inner Mongolia autonomous region of China, from 2012 to 2016. Emerg Microbes Infect. (2020) 9:263–74. 10.1080/22221751.2020.1720528 31997725 PMC7034055

[30] Cao X Li Z Liu Z Fu B Liu Y Shang Y. Molecular epidemiological characterization of *Brucella* isolates from sheep and yaks in northwest China. Transbound Emerg Dis. (2018) 65:e425–e33. 10.1111/tbed.12777 29193808

[31] Zeng J Duoji C Yuan Z Yuzhen S Fan W Tian L. Seroprevalence and risk factors for bovine brucellosis in domestic yaks (*Bos grunniens*) in Tibet, China. Trop Anim Health Prod. (2017) 49:1339–44. 10.1007/s11250-017-1331-7 28624927

[32] Liu ZG Di DD Wang M Liu RH Zhao HY Piao DR. MLVA genotyping characteristics of human *Brucella melitensis* isolated from Ulanqab of Inner Mongolia, China. Front Microbiol. (2017) 8:6. 10.3389/fmicb.2017.00006 28149294 PMC5241320

[33] Piao DR Liu X Di DD Xiao P Zhao ZZ Xu LQ. Genetic polymorphisms identify in species/biovars of *Brucella* isolated in China between 1953 and 2013 by MLST. BMC Microbiol. (2018) 18:7. 10.1186/s12866-018-1149-0 29361930 PMC5781281

[34] Cao X Li S Li Z Liu Z Ma J Lou Z. Enzootic situation and molecular epidemiology of *Brucella* in livestock from 2011 to 2015 in Qingyang, China. Emerg Microb Infect. (2018) 7:58. 10.1038/s41426-018-0060-y 29615607 PMC5882930

[35] Liu ZG Wang M Zhao HY Piao DR Jiang H Li ZJ. Investigation of the molecular characteristics of *Brucella* isolates from Guangxi Province, China. BMC Microbiol. (2019) 19:292. 10.1186/s12866-019-1665-6 31842756 PMC6916230

[36] Ferreira Vicente A Girault G Corde Y Souza Ribeiro Mioni M Borges Keid L Jay M. The study of worldwide *Brucella canis* of phylogenetic groups by comparative genomics-based approaches. China Biotechnol. (2020) 40, 38–47. 10.13523/j.cb.190705330153798

[37] Liu ZG Wang LJ Piao DR Wang M Liu RH Zhao HY. Molecular investigation of the transmission pattern of *Brucella suis* 3 from inner Mongolia, China. Front Vet Sci. (2018) 5:271. 10.3389/fvets.2018.00271 30420955 PMC6215816

[38] De-gui L Current status, opportunities and challenges of pet industry in China. Chin J Comp Med. (2010) 20:13–6. 10.3969/j.issn.1671/7856.2010.11.12.004

