# Supplementary figures and images for: ONE Health Approach to Address Zoonotic Brucellosis: A Spatiotemporal Associations Study Between Animals and Humans
Source: Front Vet Sci. 2020 Sep 2;7:521. doi: 10.3389/fvets.2020.00521 (PMC7492289; doi:10.3389/fvets.2020.00521)

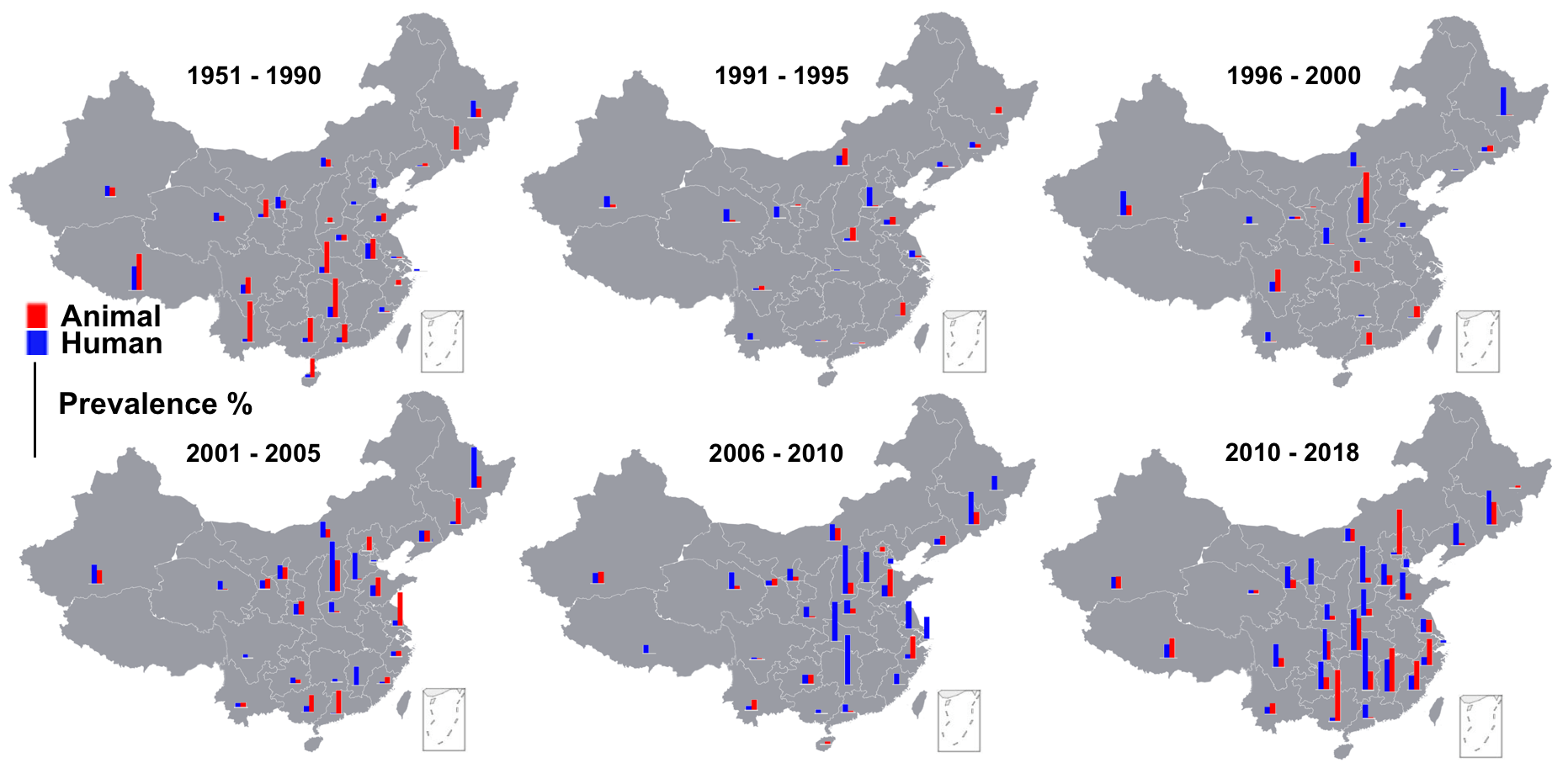

Supplement: Supplemental Figure 1 — Spatiotemporal prevalence of human and animal brucellosis in China from 1951 to 2018. [file Image_1.TIFF]
